# Towards the cell-instructive bactericidal substrate: exploring the combination of nanotopographical features and integrin selective synthetic ligands

**DOI:** 10.1038/s41598-017-16385-3

**Published:** 2017-11-27

**Authors:** Roberta Fraioli, Penelope M. Tsimbouri, Leanne E. Fisher, Angela H. Nobbs, Bo Su, Stefanie Neubauer, Florian Rechenmacher, Horst Kessler, Maria-Pau Ginebra, Matthew J. Dalby, José M. Manero, Carlos Mas-Moruno

**Affiliations:** 1 Biomaterials, Biomechanics and Tissue Engineering Group, Department of Materials Science and Metallurgical Engineering, Technical University of Catalonia (UPC), Barcelona, 08019 Spain; 2 Barcelona Research Center in Multiscale Science and Engineering, UPC, Barcelona, 08019 Spain; 3 Centre for Cell Engineering, University of Glasgow, Glasgow, G12 Scotland UK; 4 Bristol Dental School, University of Bristol, Bristol, BS1 2LY UK; 5 Institute for Advanced Study and Center for Integrated Protein Science, Department Chemie, Technische Universität München, 85748 Garching, Germany; 6 Institute for Bioengineering of Catalonia (IBEC), Barcelona, 08028 Spain

## Abstract

Engineering the interface between biomaterials and tissues is important to increase implant lifetime and avoid failures and revision surgeries. Permanent devices should enhance attachment and differentiation of stem cells, responsible for injured tissue repair, and simultaneously discourage bacterial colonization; this represents a major challenge. To take first steps towards such a multifunctional surface we propose merging topographical and biochemical cues on the surface of a clinically relevant material such as titanium. In detail, our strategy combines antibacterial nanotopographical features with integrin selective synthetic ligands that can rescue the adhesive capacity of the surfaces and instruct mesenchymal stem cell (MSC) response. To this end, a smooth substrate and two different high aspect ratio topographies have been produced and coated either with an αvβ3-selective peptidomimetic, an α5β1-selective peptidomimetic, or an RGD/PHSRN peptidic molecule. Results showed that antibacterial effects of the substrates could be maintained when tested on pathogenic *Pseudomonas aeruginosa*. Further, functionalization increased MSC adhesion to the surfaces and the αvβ3-selective peptidomimetic-coated nanotopographies promoted osteogenesis. Such a dual physicochemical approach to achieve multifunctional surfaces represents a first step in the design of novel cell-instructive biomaterial surfaces.

## Introduction

According to a projection study through 2030, the burden of primary and revision joint arthroplasties is expected to increase significantly^1^. The number of hip replacement primary surgeries will grow by 174% by 2030 in the United States (from 208,600 in 2005 to 572,000), causing a growth in revision surgeries, which are expected to double by year 2026. Thus, despite a track record of positive outcomes, orthopedic implant failure will be a major clinical concern in the future as we strive to support the aging population. To address this problem, the two leading causes of implant failure should be taken into consideration: aseptic loosening and infection, which account for 18% and 20% of revision of total knee arthroplasty, respectively^2^. Though several causes for aseptic loosening exist, poor osteointegration is one of them. This highlights the importance of developing the design of multifunctional orthopedic coatings that are simultaneously antibacterial and osteoinductive^3,4^. With a few recent exceptions^5,6^, most studies focus on either conferring cell-guiding or antibacterial properties to the surfaces, as antibacterial coatings, such as silver and antibiotics, tend to provide lower osteoblast bioactivity^7,8^.

In the past ten years nanotopography has emerged as a potent tool to tune the response of human stem cells^9–11^ and, more recently, to give antibacterial properties to the surface^12–14^. Topographical features of the surface at the nanometre scale have been shown to affect adhesion, proliferation and differentiation of mesenchymal stem cells (MSCs) and osteoprogenitor cells to osteoblasts^11,15–17^. This effect has been ascribed to the increased cytoskeletal tension, which is known to drive osteogenesis^18,19^.

Following a biomimetic rationale, nanotopographies have also been shown to be able to be tuned to have antibacterial properties. Features similar to those of bactericidal insects’ wings (the Clanger cicada (*Psaltoda claripennis*)^20,21^ and the dragonfly *Diplacodes bipunctata* ^22^) have been reproduced on the surface of artificial materials such as titanium and black silicon^12,22^. Both insects’ wings present high aspect ratio surface nanometric features that have been demonstrated to be bactericidal. The needle-like features were found to effectively kill bacteria by imposing high deformational stresses on their membranes (leading to rupture or piercing)^20^. Such intrinsic bactericidal properties are particularly interesting for application in biomaterial surfaces since they are devoid of the limitations of numerous antibacterial coatings, such as silver- or antibiotic-releasing coatings, i.e. the initial burst release, which can be cytotoxic to cells, the difficulty in controlling the release profile, the risk of developing antibiotic resistance and the limited lifespan, given the decreasing concentration of the released species^4^.

Although antibacterial nanotopographies with the capacity to positively stimulate eukaryotic cells have been reported^23–27^, the high aspect ratio nanotopographies are often not optimal for eukaryotic cell-instructive purposes (e.g. osteointegration) as they are quite different from the ones cited in the literature to promote osteogenesis^11,15–17^ and could even lead to a reduction in the cell adhesive properties of the surface. A viable solution is chemical functionalization to anchor cell receptor binding molecules on the surface in order to promote better human MSC (hMSC) attachment and development of increased cytoskeletal tension required for osteospecific differentiation and hence bone integration. A family of biomolecules derived from bone extracellular matrix (ECM), from full-length proteins^28^ or recombinant protein fragments^29^, to small peptidic sequences^30–34^, and peptidomimetic ligands^35^, have been used to create MSC-instructive substrates and thus can potentially be used to rescue the cell-instructive capacity of antibacterial nanotopographies.

**Figure 1:**
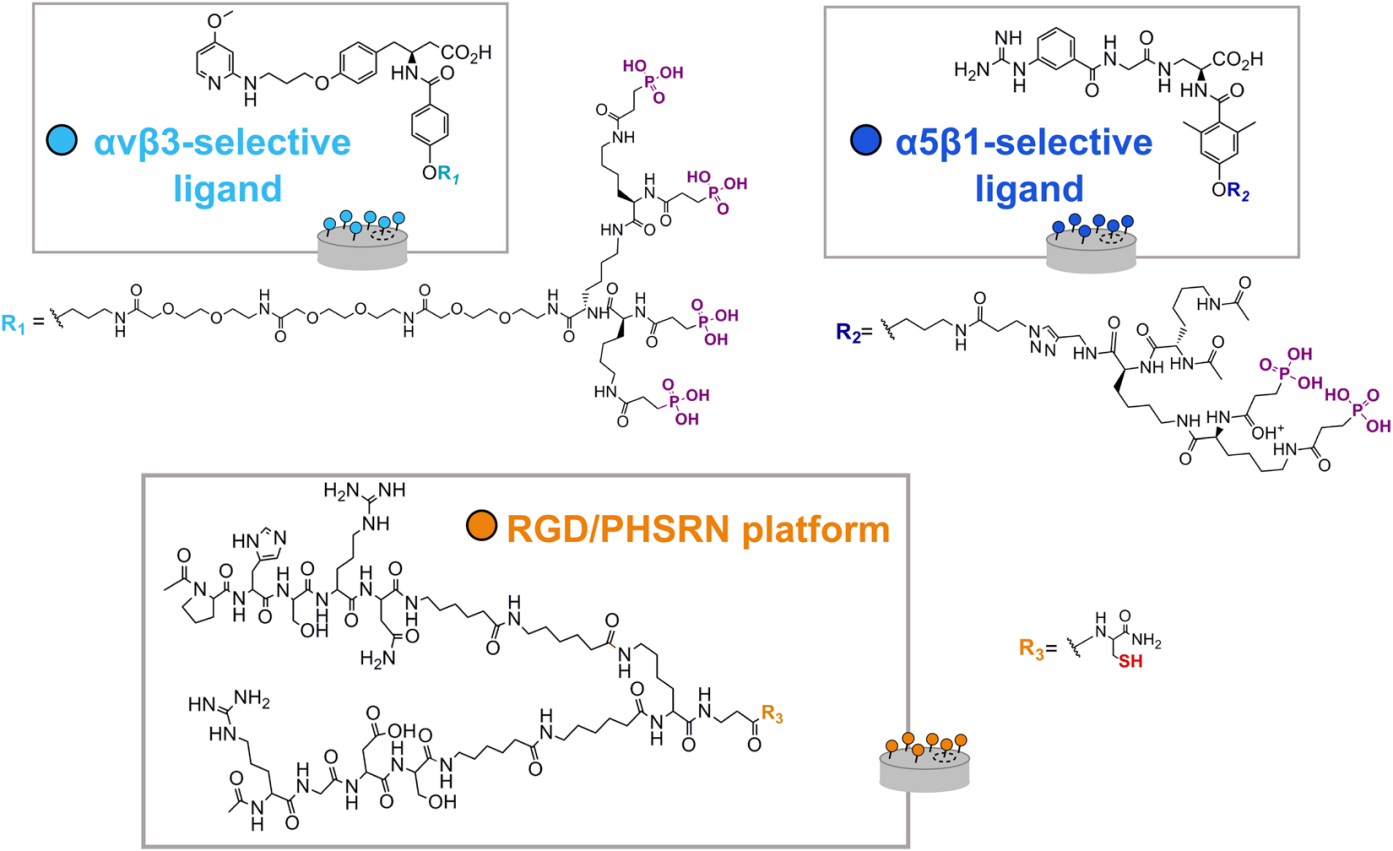
Chemical structure of the integrin-binding ligands: αvβ3-selective peptidomimetic (light blue), α5β1-selective peptidomimetic (dark blue), and RGD/PHSRN platform (orange). Phosphonic acid anchors are highlighted in purple and thiol anchor is highlighted in red.

Herein we present a combined approach that merges high aspect ratio nanotopographical cues with the chemical grafting of integrin-binding peptidic ligands to engineer a bactericidal and osteoinductive titanium (Ti) surface. Two different nanotopographies generated by hydrothermal treatment were tested. Three different peptidic ligands were coupled to the topographies. Two were integrin-selective peptidomimetic molecules, selective for integrin αvβ3 or α5β1 respectively^35–37^, which were previously shown to mediate integrin-specific cell adhesion^35,36^ and to promote osteointegrative events on Ti^38^, and the third a double-branched peptidic ligand containing the RGD and PHSRN sequences from fibronectin (FN), with the capacity to improve osteoblast-like and MSC behavior on the surface of Ti^30,34^. The chemical structure of the three coating molecules is shown in Fig. 1. These types of ligands have shown significant advantages compared to the use of ECM native proteins and short synthetic peptides in terms of integrin-binding activity, specificity and stability. This report is the first example describing their application to coat bactericidal surfaces.

MSCs, osteoblast forming cells of the bone marrow that typically come into contact with the areas of e.g. hip/knee prosthesis where bone bonding is required, were used to test osteogenesis. *Pseudomonas aeruginosa* was used to test bacterial attachment as this bacterium is Gram–negative and thus should be easier to kill due to lack of a thick peptidoglycan layer within its cell wall; it is also a biofilm forming pathogen that can lead to implant failure in the clinical setting.

## Results

### Surface characterization

**Figure 2:**
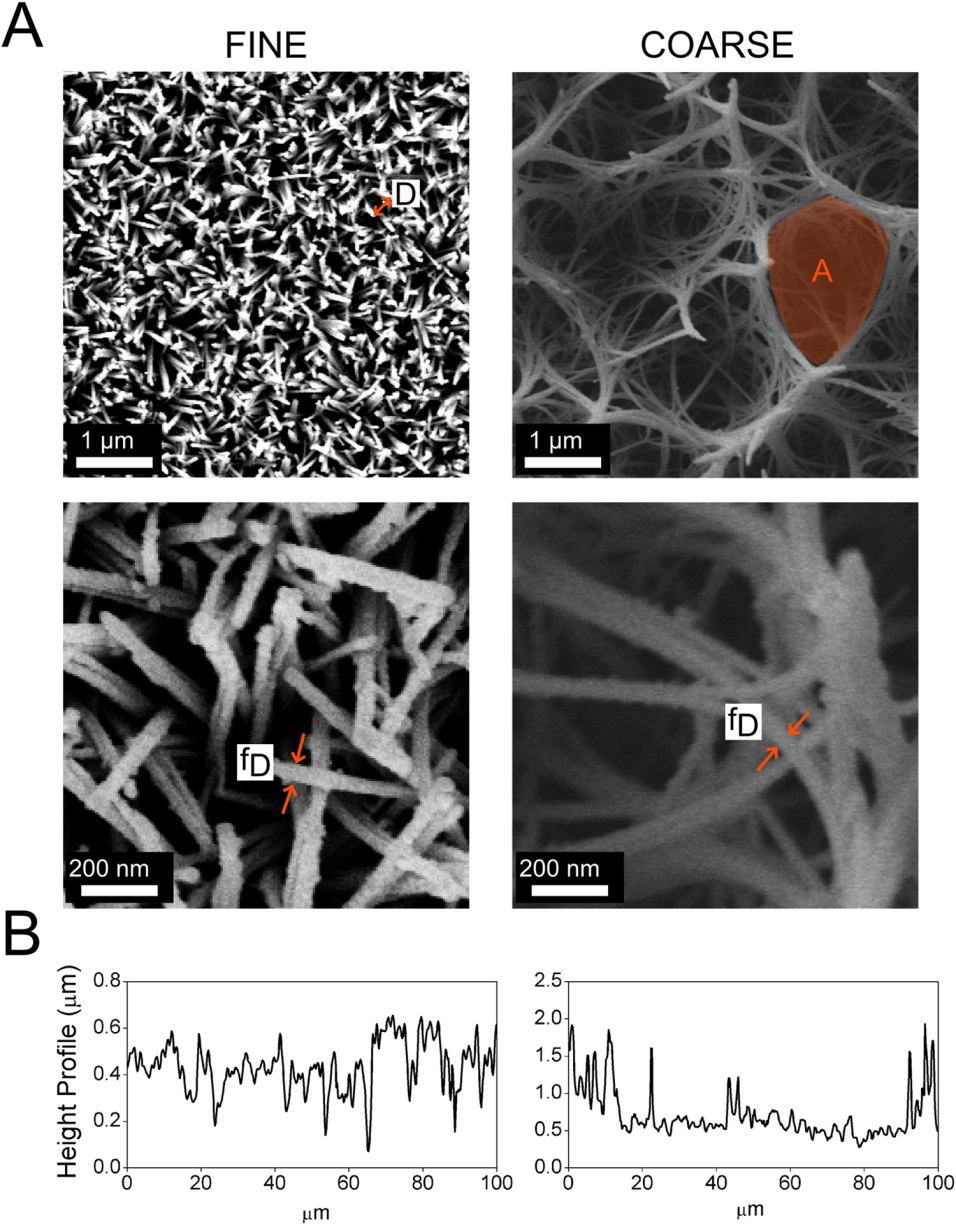
(**A**) SEM images of the nanotopographies. The labels tip-to-tip distance - D, “pocket” area - A, fibre diameter - f_D_ refer to the measured geometrical features of the nanostructure in Table 1. (**B**) Height profile of the FINE (left) and COARSE (right) topographies.

Two nanotopographies have been generated by applying a hydrothermal treatment to Ti samples using different reaction times. As shown in Fig. 2A, the length of the fibres increased with reaction time: the 2 h treatment generates homogeneous fine spike-like structures (FINE); when reaction time is increased to 3 h, these structures grow in length and merge to form much bigger pocket-like structures on the surface (COARSE). The hydrothermal treatment conditions and the geometrical features of these structures, obtained from the SEM image analysis, are summarized in Table 1 and the height profile is reported in Fig. 2B. Further topographical values are available in Supplementary Table S1.

**Table 1:** Hydrothermal treatment conditions and geometrical features of the nanotopographies.

Nanotopography	Geometrical feature		Treatment conditions
FINE	D	(171.3 ± 48.3) nm	240 °C/2 h
	f_D_	(34.0 ± 6.5) nm	
COARSE	A	(2.90 ± 1.80) μm^2^	240 °C/3 h
	f_D_	(7.78 ± 2.56) nm	

The labels (tip-to-tip distance - D, “pocket” area - A, fibre diameter - f_D_) refer to the highlighted features of the structures in Fig. 2A. Values are reported as mean ± SD.

The binding of the molecules was performed according to well-established protocols in our laboratories. Anchoring of the platform (P) to control (FLAT) Ti via silanization with (3-aminopropyl)triethoxysilane (APTES) has been characterized by us in previous studies^34,38^ and corroborated here by X-ray photoelectron spectroscopy (XPS) (Supplementary Table S2). The presence of the silane was characterized by the Si 2p signal and the subsequent deposition of the biomolecule by the respective increase in the C 1s and N 1s signals (typical indicators of the peptidic backbone) and decrease in the detection of the titanium oxide layer, Ti 2p and O 1s, in agreement with the literature^34,38^. Applying this protocol to the nanotopographies resulted in very similar results and indicated the successful anchoring of the platform to the substrates (Table S2). Chemisorption of the peptidomimetics (V3, 51) was achieved by direct binding of the phosphonic acid groups to Ti oxide^39–41^. XPS data confirmed both ligands were immobilized to the surfaces, although the V3 ligand seemed to be bound to a slightly higher extent than the α5β1-selective one (normalized nitrogen ratios are shown in Table S2).

### SEM observation of cell attachment to the topographies

**Figure 3:**
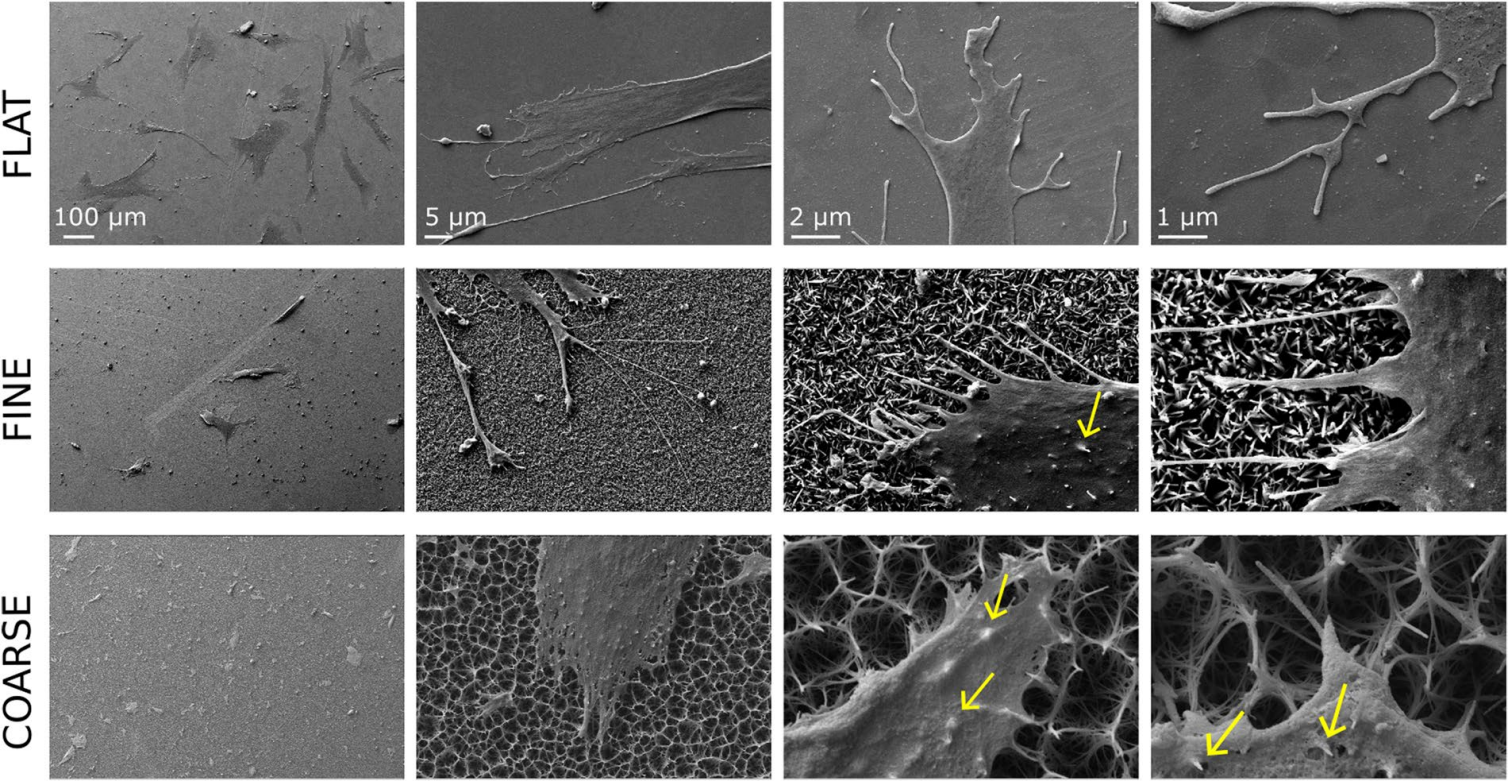
SEM images of hMSCs attaching on the flat Ti and Ti nanotopographies. Cells interacted with the nanofeatures as can be seen by retraction fibre terminations and membrane indentations. This effect was most notable on the COARSE topography. Each column of images has the same magnification.

Low magnification images presented in Fig. 3 show that cells appeared well spread on the FLAT control surfaces. However, the cells appeared more rounded on both the FINE and COARSE topographies. Retraction fibres (long/branched) and filopodia (short/straight) were seen on all control and test sample surfaces with cells observed interacting with topographical features (Fig. 3). Also notable were impressions of the underlying topography in the cell membranes (Fig. 3).

### Effect of topography and ligand presentation on the number, area, and circularity of attached cells

**Figure 4:**
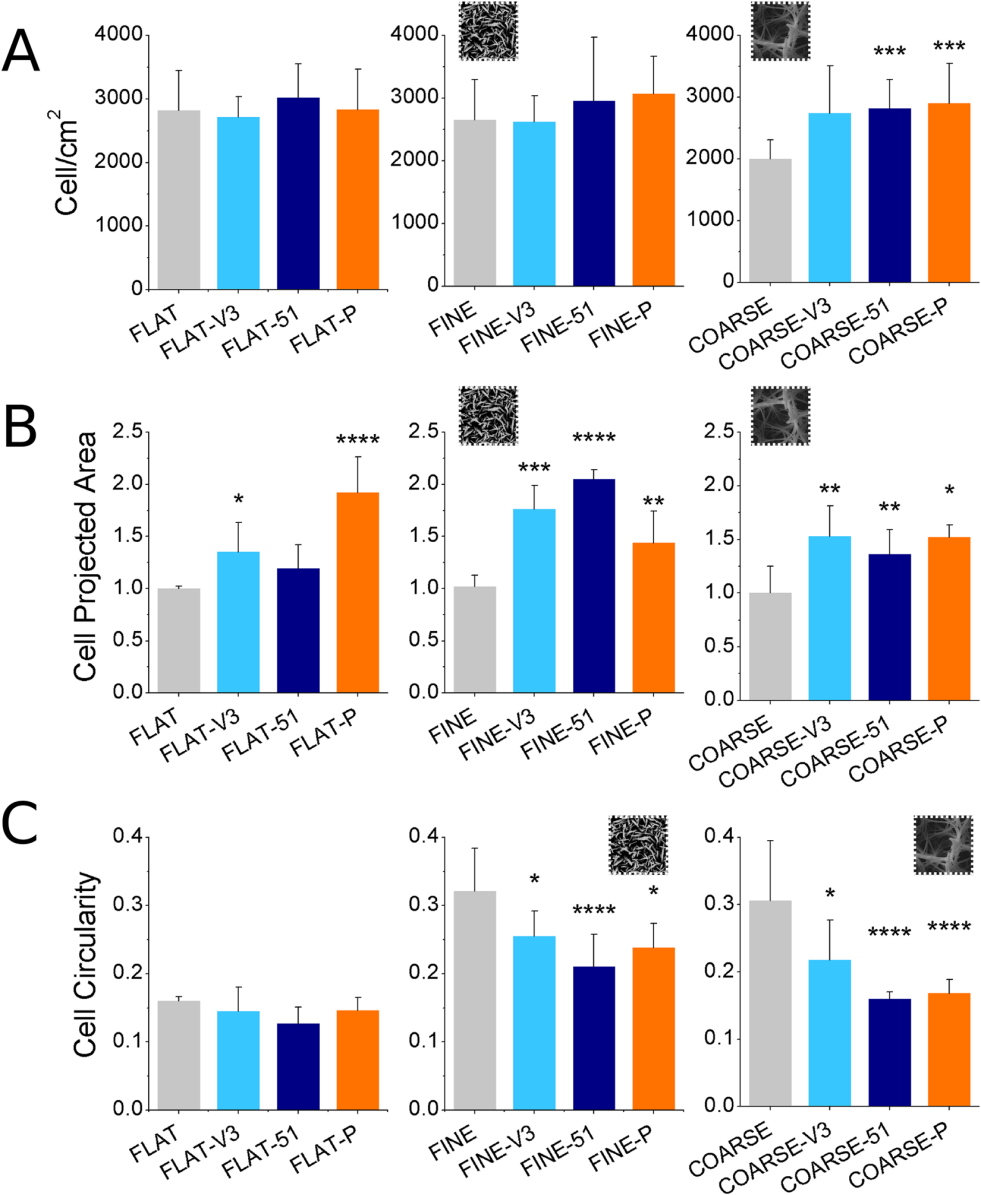
Number of attached cells (**A**), cell projected area (**B**), and cell circularity (**C**) on the FLAT (first column of graphs), FINE (second column of graphs), and COARSE (third column of graphs) surfaces with or without biomolecule coating. Cell projected area has been normalized to the uncoated condition in each graph (FLAT, FINE, and COARSE, respectively). Number of attached cells was not affected by the biomolecules on FLAT and FINE, while more cells adhered to the biomolecule-coated COARSE substrates. Cell area was significantly increased in the presence of the biomolecule on all three topographies. While low circularity was observed on all FLAT conditions, the biomolecule-coated FINE and COARSE surfaces presented lower circularity compared to the respective uncoated topography. *p < 0.05, **p < 0.01, ***p < 0.001, ****p < 0.0001 vs. uncoated condition (FLAT, FINE and COARSE, respectively).

For all cellular experiments including biomolecules, cell seeding was performed in serum-free conditions. This allows cells to attach to the surface mainly via the receptor-binding ligands and avoids non-specific interactions with serum proteins. Characterization of short-term cell response to the functionalized topographies was performed by incubating hMSCs for 24 h on the substrates and using fluorescent staining of nuclei and actin cytoskeleton. The number of cells attached to the FLAT and FINE Ti samples was not significantly affected by the presence of the synthetic ligands (Fig. 4A). Nonetheless, on the COARSE topography, where fewer cells adhered in the uncoated condition compared to the FLAT and FINE surfaces, the presence of the synthetic ligands provided an increase in cell number, which was statistically significant in the case of the α5β1-selective mimetic (COARSE-51) and the RGD/PHSRN platform (COARSE-P).

Although the presence of the receptor-binding ligands only affected attachment on the COARSE surface, hMSC projected area was increased by the addition of the biomolecules on all topographies (Fig. 4B). On the FLAT surfaces, this increase was statistically significant only for the αvβ3-selective mimetic (FLAT-V3) and the RGD/PHSRN platform (FLAT-P). However, on the FINE and COARSE topographies, all biomolecules generated a significant increase in area compared to the uncoated sample of the same topography. Though cells appeared smaller on FINE and COARSE uncoated topographies compared to FLAT, no statistically significant difference in cell area was observed comparing the three uncoated topographies (see Supplementary Fig. S1). Finally, quantification of cell circularity showed that while hMSC shape was unaffected by the presence of the peptide on the FLAT surface (Fig. 4C), when grafted to the topographies all the ligands caused significant reduction in circularity.

**Figure 5:**
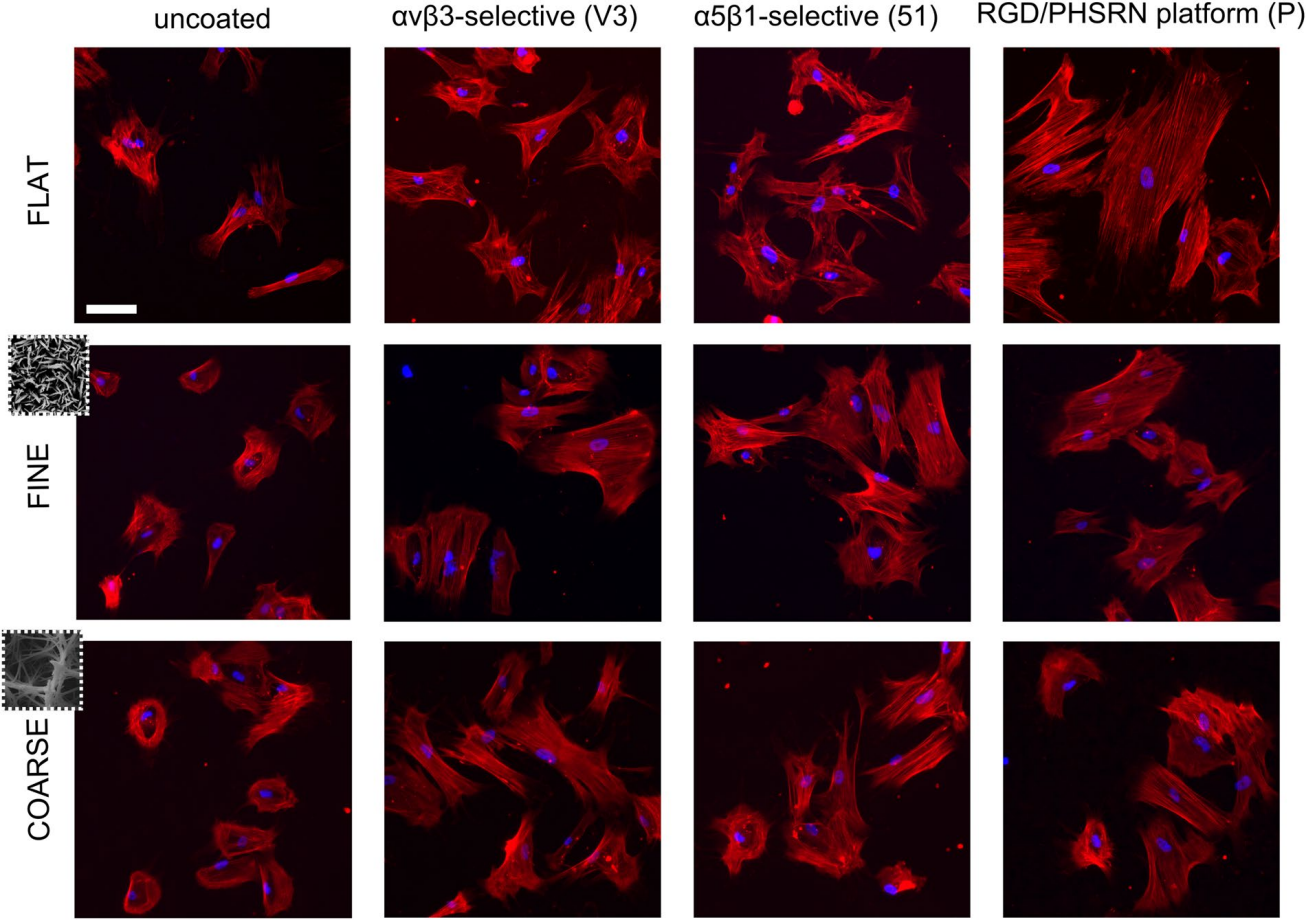
Immunostained actin fibers and DAPI-stained nuclei. Cell seemed to spread more on the biomolecule-coated nanotopographies, compared to their uncoated control. A decrease in circularity was also evident on the COARSE and FINE substrates presenting integrin-binding ligands. Scale bar = 100 μm.

Actin cytoskeletal staining (Fig. 5) confirmed these measurements with little change in cell shape observed on FLAT control, but notable changes from smaller, more rounded cells to larger, more polygonal cells observed with all biomolecule modifications on the topographies.

### Focal adhesion morphology in response to topographies and coatings

**Figure 6:**
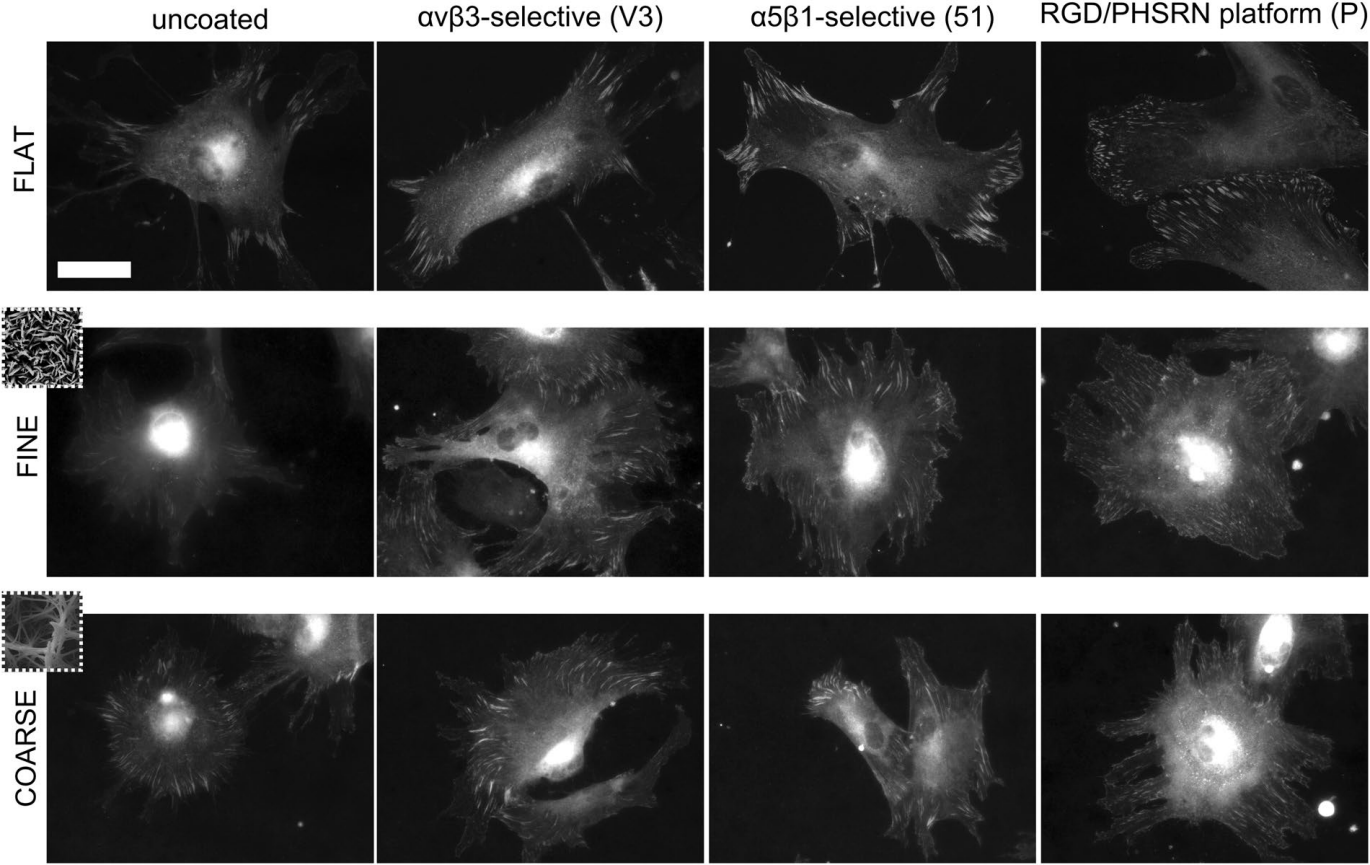
Immunostaining of vinculin. Fluorescence intensity of vinculin adhesion sites on the biomolecule-coated substrate was increased when compared to the uncoated controls. Scale bar = 50 μm.

**Figure 7:**
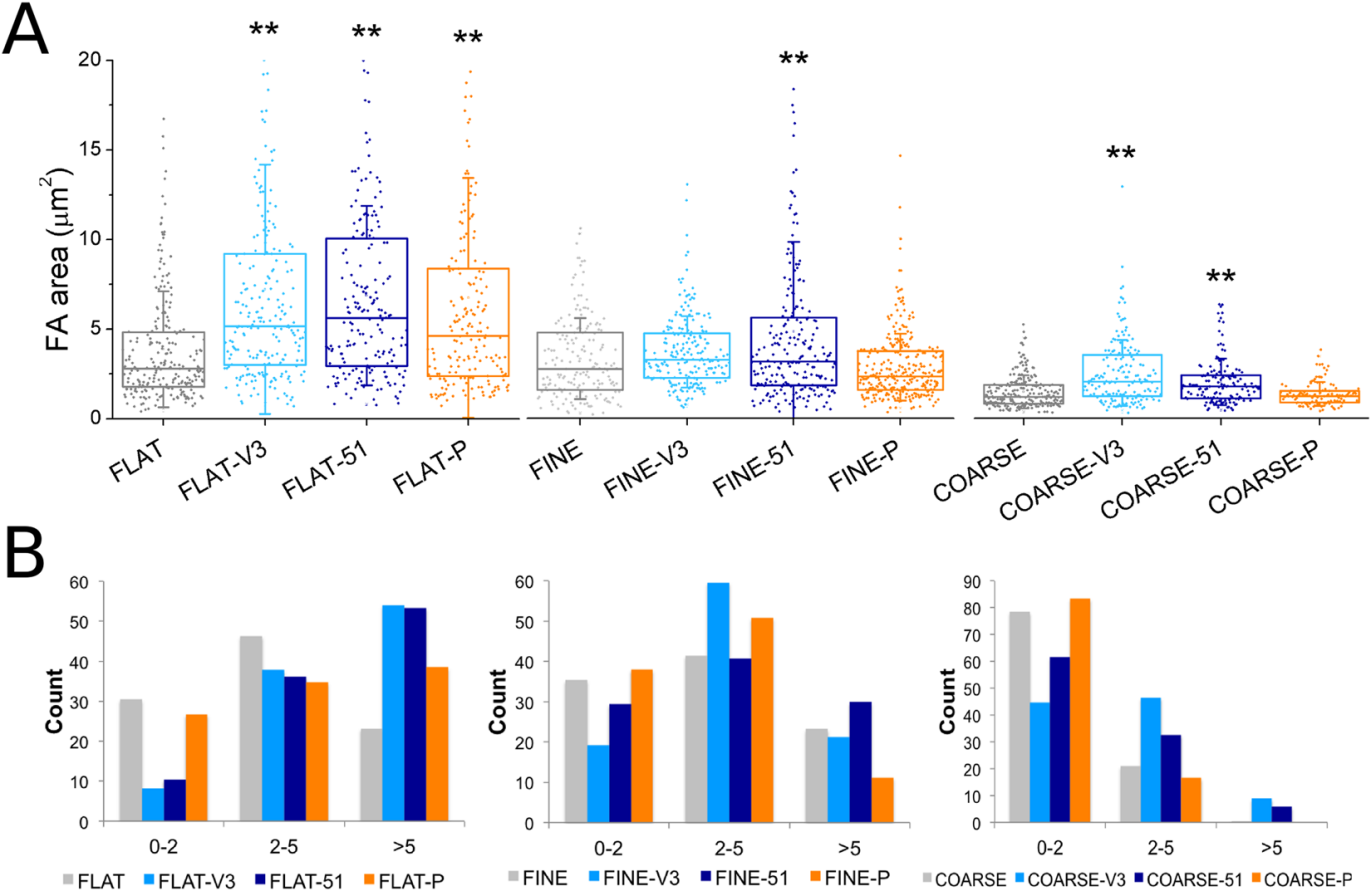
(**A**) Focal adhesion area on FLAT, FINE, and COARSE topographies. Passing from the smooth Ti to FINE and COARSE surfaces, FA area was reduced. However, upon grafting biomolecules, the dimension of the FA was increased compared to the uncoated condition of each topography. **p < 0.01 vs. uncoated condition (FLAT, FINE and COARSE, respectively). (**B**) Adhesion length binned by focal complexes (0–2 µm), focal adhesions (2–5 µm) and super mature adhesions (>5 µm).

hMSCs cultured for 24 h on the coated and uncoated Ti samples were immunostained to visualize vinculin, a protein commonly used to label focal adhesions (Fig. 6). Imaging suggested that cells increased adhesion on all the biofunctionalized surfaces (both FLAT and nanotopographies). By means of image analysis, the dimensions of focal contacts were quantified (Fig. 7). Focal contact area appeared to be influenced by both the topography and the chemistry of the substrate. The effect of both nanotopographies (FINE and COARSE) was to produce an overall reduction in the distribution of focal adhesion (FA) area, compared to the FLAT surface. However, the effect of the biomolecules was to generate a shift towards larger focal adhesions, compared to the uncoated topographies (Fig. 7A). This increase in FA area was statistically significant on the FINE-51, COARSE-V3 and COARSE-51 surfaces, compared to their uncoated controls (FINE and COARSE, respectively).

When length is considered and binned by focal complexes (0–2 µm), focal adhesions (2–5 µm) and super mature adhesions (>5 µm)^42^ changes in adhesion could be further described. On the FLAT controls, a clear shift in adhesion length from a preponderance of focal complex formation to that of super mature adhesion formation was noted (Fig. 7B). For the FINE and COARSE substrates a similar but reduced shift in trend was also noted for V3 and 51 biofunctionalization (Fig. 7B). Notably, for the COARSE substrates, super mature adhesions were only evident on the COARSE-V3 and 51 modifications (Fig. 7B).

### Expression of osteogenic genes

**Figure 8:**
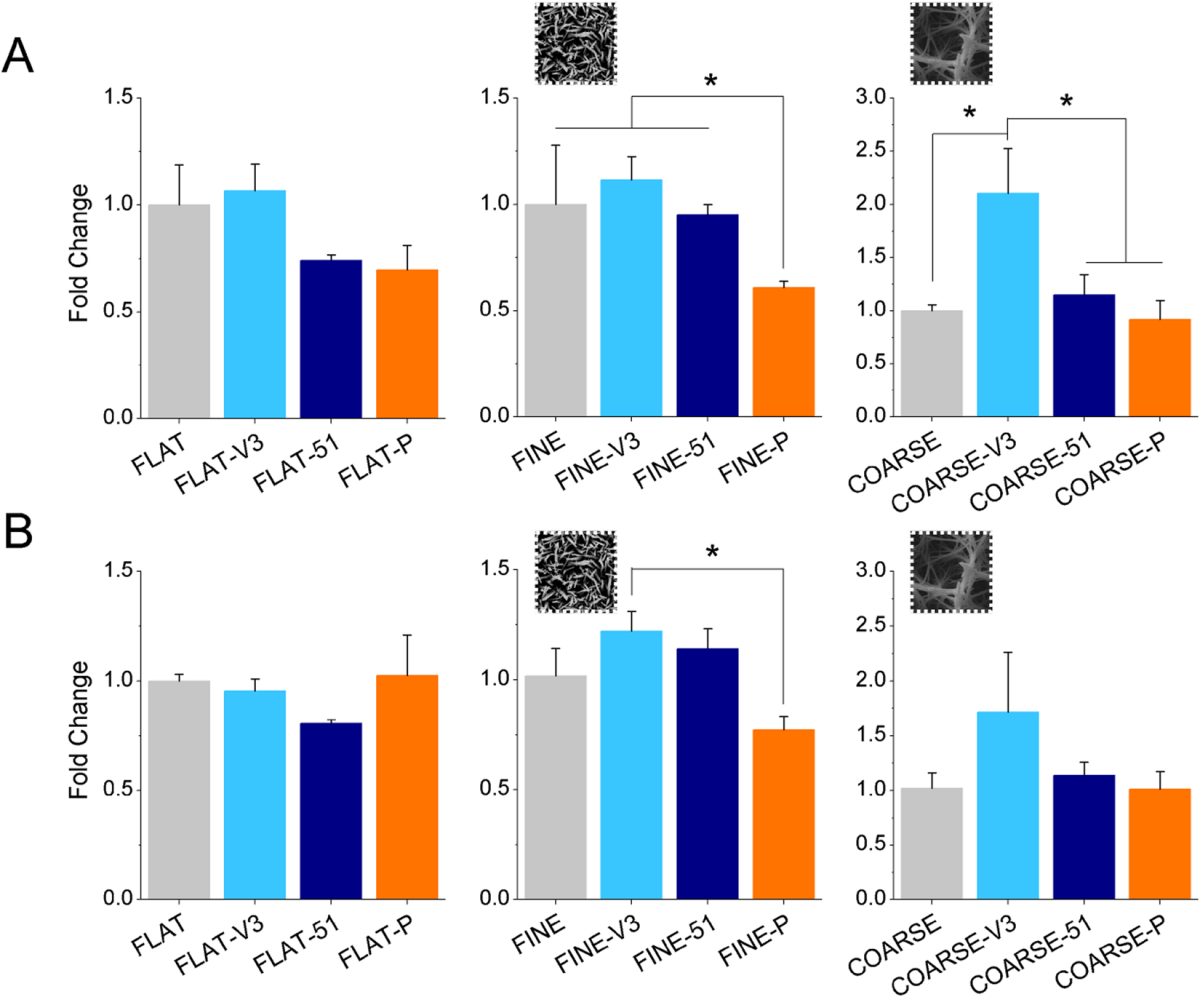
Expression of the osteogenic markers osteocalcin (**A**) and osteopontin (**B**). A clear trend towards higher marker expression on the αvβ3-selective peptidomimetic (V3) was visible, though statistically significant only on the COARSE topography. Data have been normalized to the uncoated condition of the respective topography. *p < 0.05 vs. uncoated condition (FLAT, FINE and COARSE, respectively).

Quantitative PCR analysis of gene expression was carried out after 21 days of incubation in basal medium on the uncoated and biomolecule-coated topographies. The expression of the late osteogenic markers OCN and OPN was evaluated in order to monitor the progression of hMSCs along the osteoblastic lineage (Fig. 8). The trend for expression of both genes was very similar on all topographies in that the αvβ3-selective peptidomimetic stimulated the highest expression of both OCN and OPN on FLAT, FINE and COARSE Ti topographies, while the α5β1-selective peptidomimetic and the RGD/PHSRN peptidic platform did not change gene expression significantly. The trend was most notable on the V3 topographies where functionalization had reversed the rounded morphology caused by the high aspect ratio features. The increase caused by the αvβ3-selective peptidomimetic was, however, only significant on the COARSE topography that had, when uncoated, provided the greatest rounding up of the MSCs.

### Topography affects bacterial attachment

**Figure 9:**
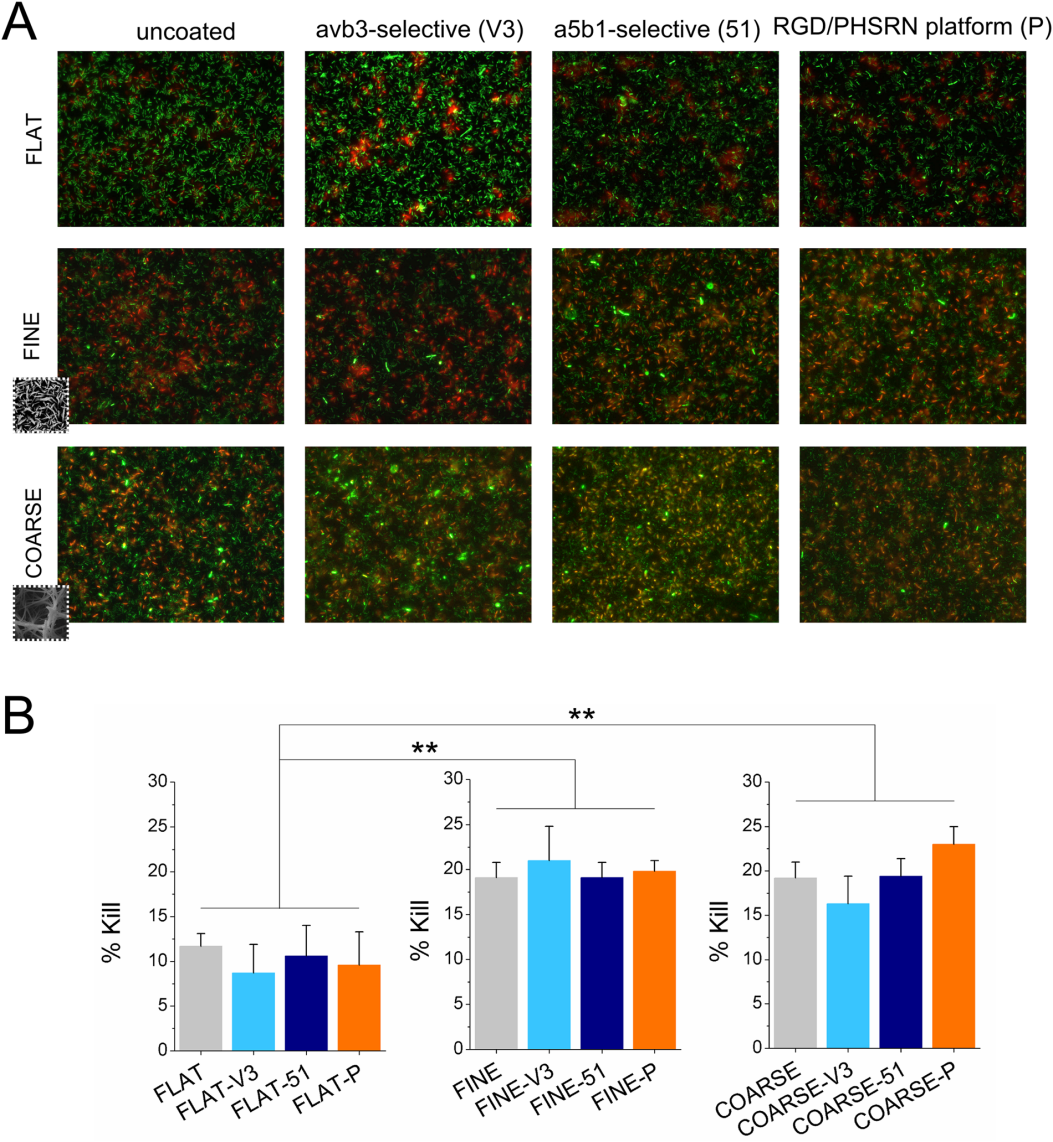
Representative fluorescence microscopy images (**A**) of *P. aeruginosa* stained with Live/Dead viability stain and (**B**) percentage of dead cells. Live cells are stained green, while dead cells appear red. Increase in the % kill was observed on both nanotopographies, compared to the FLAT Ti surface. No effect of the biomolecules was visible. **p < 0.01 vs. uncoated condition (FLAT, FINE and COARSE, respectively).

In order to check the effect of topography and ligands on the bactericidal properties of the surfaces, bacterial attachment (*P. aeruginosa*) assays were carried out on the substrates. Compared to the polished Ti surfaces (FLAT), both topographies (FINE and COARSE) presented a significantly increased number of dead cells (Fig. 9). The kill level for both nanotopographies was about 20% after 1 h. No effect on bacterial killing was seen from the grafted ligands (Fig. 9B).

## Discussion

A range of metallic materials, including Ti and its alloys, have been optimized to serve as biomaterials for joint replacement implants^43^. However, premature failure, mainly due to aseptic loosening or infection, remains prevalent. Implants should thus, ideally, allow integration with the surrounding tissues through osteoinduction of bone marrow MSCs and reduce bacterial colonization to prevent implant-related infection or chronic biofilm formation^4^.

With the aim of producing a multi-functional Ti surface that is both osteoinductive and antibacterial, we proposed merging two classical surface functionalization strategies, namely topographical and chemical modification. The hydrothermal treatment described in this study allows for the generation of Ti substrates with nanoscale, high aspect ratio, topographical features that can be produced over large areas and on complex, 3D surfaces. The rationale behind the generation of such topographies is derived from biomimesis of natural bactericidal surfaces, such as the wings of the Clanger cicada (*Psaltoda claripennis*)^12,20,21^ and the dragonfly *Diplacodes bipunctata* ^22^. However, as was again shown here, high aspect ratio features tend to reduce cell/MSC adhesion^11^, and this limits their usefulness in terms of improving osteointegration. Thus, we combined here for the first time the high aspect ratio topographies with recently designed low molecular weight integrin-binding molecules with affinity for αvβ3 or α5β1 receptors; both integrins have been described to mediate important events in bone biology^35,44^. In this regard, previous studies from our group already reported stimulation of osteoblast-like and MSC attachment with such ligands^30,34,38^. As previously introduced, these high affinity molecules circumvent the limitations of classical ECM-derived approaches, such as RGD peptides, which show relatively low integrin-binding profiles and poor stability, and therefore represent ideal candidates for the bioactivation of the nanotopographies.

As observed in the lowest magnification SEM images, fewer hMSCs attached and spread on the nanotopographies compared to the FLAT, polished Ti surface. Similarly, immunostaining of actin cytoskeleton revealed that cell shape was more circular on both the FINE and COARSE topographies compared to the FLAT surface. However, adhesive capacity of the nanotopographies was rescued after grafting integrin-binding peptides. This was evidenced by a significant increase in cell area and a decrease in cell circularity, which can be proposed to correspond to biochemical interactions with the substrates via integrin receptors and therefore indicates a better interaction with the metal. Importantly for this study, the effect of the biomolecules was more evident on the low adhesion nanotopographies than on the polished control. Though few studies combined topographical stimulation and biochemical signals, an enhancing effect in terms of cell attachment was previously observed on RGD-functionalized nanoporous alumina membranes^45^ and sandblasted Ti6Al4V disks^40^.

Study of cell adhesions yielded similar results. In the 1980’s it was noted that adhesions could adopt a dot or dash shaped morphology^46^ and later this has been further subdivided into focal complex (small, 1–2 µm, transient adhesions), focal adhesions (larger, 2–5 µm, stable adhesions) and super mature adhesions (or fibrillar adhesions, large stabilising adhesions required to support the contractile forces generated during osteogenesis^18,19,42,47^). Data showed that addition of the integrin binding molecules increased adhesion area on all surfaces. This was most notable on the COARSE-V3 and COARSE-51 surfaces as for these larger features only these modifications permitted formation of super mature adhesions.

Looking at osteospecific marker genes osteocalcin and osteopontin, in the main only small trends were noted. However, for the COARSE topography coated with the αvβ3-selective peptidomimetic, where super mature adhesions had been allowed to establish compared to the uncoated control, increased expression of the transcripts was noted. The positive effect of this integrin subtype on osteogenic differentiation of osteoblasts and MSCs has been previously reported^48,49^. Integrin subtype αvβ3 is selective for vitronectin, which has been implicated in increased adhesion bridging compared to FN (which preferentially binds integrin α5β1)^50^. Potentially, such bridging would facilitate increased adhesion between topographical features^11^. From all of these observations on cell spreading, adhesion and differentiation, it seems that both the peptidomimetic and FN-inspired α5β1-selective surfaces are less effective in stimulating osteogenesis on these substrates.

Finally, nanotopography-related bactericidal properties of the samples were tested with *P. aeruginosa* attachment assays. Similar surfaces were previously reported to be bactericidal^12^, however, such features have not been tested in the presence of cell adhesion ligands and it is critical that we only target mammalian cell adhesion without affecting bacterial kill. As expected, both FINE and COARSE nanotopographies were more effective than flat Ti in inducing bacterial death due to the mechanical effect of their high aspect ratio nanofeatures. Coating of the substrates with the integrin-binding ligands did not affect the bactericidal properties of the nanostructured substrates, thereby indicating that the antibacterial effect is caused by the topography of the surface, rather than by biochemical signals. This mechanical bactericidal effect has been observed before on artificial surfaces presenting similar bio-inspired nanotopography^22^.

Nanotopographies capable of achieving both antibacterial effects and eukaryotic cell adhesion would be desirable for medical implant applications^23–27^. However, topographical features alone will always be limited in terms of bioactivity, since the surfaces with maximum antibacterial potential might not be favorable for the optimal osteoinductivity, or vice versa. The biofunctionalization of nanotopographies with chemical coatings offers the possibility to introduce a wide range of biological activities by means of very diverse biochemical cues, including osteogenic signals, growth factor derived peptides, mineralization domains or biofunctionalities required for the growth and/or repair of different tissues. This flexibility and range of applications would be extremely hard to achieve by merely topographical changes.

In this regard, biofunctionalization of high aspect ratio nanotopographical features with integrin-binding molecules is a viable method to rescue compromised cell adhesive functions while maintaining antibacterial properties. This has been shown here for the first time using a synthetic FN-mimic combining the RGD and PHSRN motifs and two integrin-specific RGD-based peptidomimetics, which significantly improved the adhesion of MSCs to the nanotopographies in terms of spreading, reduced circularity and focal adhesion formation. The use of synthetic molecules for such purposes holds great promise, as it overcomes the limitations and regulatory issues described for cell- and protein-based therapies^51^. Peptidomimetic molecules are particularly attractive for *in vivo* applications because they are highly stable to changes in pH and temperature, and, notably, to enzymatic cleavage^35^. Moreover the use of phosphonic acids as anchoring units has shown to be suitable to resist friction forces during implantation^52^. According to the results of the present study, the αvβ3-selective mimetic might be the most promising choice for clinical applications, as it appeared to be more osteogenic than the other molecules. Nevertheless, further experimental testing would be needed to draw a definitive conclusion.

## Conclusions

The design of multifunctional coatings has increasing relevance, especially to solve multiple issues, such as infection and impaired osteointegration of joint-replacement implants. We acknowledge that the kill level for our multifunctional surfaces was only ~20% after 1 h of bacterial culture. However, previous work with similar substrates has shown bacterial killing to increase with time (from ~25% at 1 h to ~55% at 18 h)^23^. We also note that using pure materials properties, such as topography, to kill bacteria is a very new avenue of research. We further accept that a remarkable enhancement of osteogenesis was not observed. Again, however, we believe that achieving better MSC adhesion to such spiky features and recovering expression of osteospecific transcripts helps us to take a step forwards in the ‘big ask’ of designing implants that favour eukaryotic cells over prokaryotic cells. This line of research is important considering the threat of the post-antibiotic era and the increase in prevalence in complications arising from surgery this will bring. We also foresee that the combination of topographical features with chemical coatings might be expanded to design surfaces with multiple biological functions for a wide range of applications.

## Methods

### Ti substrate preparation

Ti disks were prepared from a 0.9 mm thick ASTM grade 1 Ti sheet (Ti metals Ltd, UK). For the smooth control surfaces (denoted by FLAT), disks were polished to a mirror image and ultrasonically cleaned in water and ethanol. Nanotopographies were generated by an alkaline hydrothermal process, as previously described^12^. In brief, Ti disks were immersed in 1 M NaOH in a polytetrafluoroethylene (PTFE) lined steel vessel (Acid Digestion vessel 4748, Parr Instrument Company, USA) at 240 °C for either 2 h (denoted by FINE) or 3 h (denoted by COARSE). The vessel was removed after each time point from the oven and allowed to cool to room temperature (RT). The samples were rinsed in water and ethanol, sequentially, and then heat-treated at 300 °C for 1 h. To convert the sodium titanate layer generated during the process to TiO_2_, samples were immersed in 0.6 M HCl for 1 h, rinsed in water and ethanol, and finally heat treated at 600 °C for 2 h.

### Ligand conjugation to the metallic surface

All samples were preliminarily cleaned with nitric acid (1 h, RT) and rinsed with water, ethanol and acetone. Previously synthesized^30,36,37^ integrin-selective molecules (Fig. 1) were covalently attached to the surface by dissolving the molecules in phosphate buffered saline (PBS) at 100 μM, and then depositing 100 μL of these solutions overnight on Ti disk surfaces at RT. The two integrin-selective peptidomimetics were directly bound to Ti oxide via the anchoring phosphonate groups. The efficiency and stability of this coating method was proved in previous reports^39–41^ and characterized by the percentage of nitrogen using XPS (SPECS Surface Nano Analysis system GmbH, Berlin, Germany). The αvβ3-selective peptidomimetic was labeled V3, while the α5β1-selective one was labeled 51. Alternatively, the peptidic RGD/PHSRN platform (labeled as P), which has a thiol group as anchoring group, required an extra step of silanization. In that case, Ti disks were exposed to silane vapor (APTES) in a vacuum chamber and kept at 100 °C for 30 min. Straight after silanization, the crosslinking agent N-succinimidyl-3-maleimidopropionate (SMP, 7.5 M in N,N-dimethylformamide (DMF)) (Alfa Aesar, Karlsruhe, Germany) was coupled by immersing the disks in DMF under agitation for 1 h at RT and rinsing with DMF, distilled water, ethanol and acetone. The peptidic platform was then anchored following the same protocol as the peptidomimetics molecules (100 μL drop, 100 μM in 6.5 pH PBS). The dissolving buffer for the platform is slightly acidic to prevent disulfide bond formation. The binding of the molecule was analyzed by XPS. Further detailed characterization of this protocol has been reported elsewhere^34,38^. Experimental conditions were labeled according to the topography and the peptidic ligand grafted, such that FINE-V3 corresponds to the FINE topography coated with the αvβ3-selective peptidomimetic. Prior to the biological assays, all surfaces were sterilized by immersion in 70% (v/v) ethanol for 30 min followed by rinsing three times with sterile PBS. All reagents were from Sigma-Aldrich, unless otherwise stated.

### Surface topography characterization

Ti topographies were mounted onto stubs and sputter coated with gold (high resolution sputter coater, Agar Scientific) for analysis on a Zeiss Sigma FE-SEM microscope. Image analysis was done with Fiji/Image-J package^53^, and 50 values per condition were used to calculate surface geometric features. Roughness parameters for all topographies were obtained by white light interferometric microscopy (Wyko NT9300 Optical Profiler, Veeco Instruments, New York, NY, USA) in vertical scanning interferometry mode. Data analysis was performed with Wyko Vision 4.10 software (Veeco Instruments).

### Cell culture

hMSCs (Promocell, Germany) were cultured in Dulbecco’s Modified Eagle Medium (DMEM) supplemented with 10% (v/v) fetal bovine serum (FBS), 200 mM L-glutamine (Invitrogen), 100 mM sodium pyruvate, 1% MEM NEAA (Gibco) and antibiotics (6.74 U/ml Penicillin-Streptomycin, 0.2 µg/ml Fungizone) at 37 °C in humidified atmosphere containing 5% (v/v) CO_2_. Culture medium was changed twice a week, and cells used between passage 1 and 2 at the concentration of 5000 cells/well. For the PCR analysis 10000 cells/well were seeded. All reagents were from Sigma-Aldrich, unless otherwise stated.

### SEM observation of cell morphology

After seeding hMSCs in serum-free conditions on Ti surfaces, 1% (v/v) FBS medium was added 6 h later to guarantee cell survival and cells kept in culture for 3 days. When the incubation time was over, samples were rinsed once with PBS, to remove floating cells, and remaining cells fixed in 2% glutaraldehyde in 0.2 M cacodylate buffer for 1 h at 4 °C. Ti disks were then immersed in 20, 40, 60, 80 and 100% (v/v) ethanol, and 25, 50, 75 and 100% (v/v) hexamethylsilizane in ethanol. Afterwards, Ti disks were mounted onto a stub and sputter coated with gold (high resolution sputter coater, Agar Scientific) for analysis on a Zeiss Sigma FE-SEM microscope. All reagents are from Sigma-Aldrich, unless otherwise stated.

### Cell attachment and immunostaining

Cells were seeded and kept in serum-free medium for 6 h. The medium was then replaced with 1% (v/v) FBS medium and kept in culture for 18 h. At harvest (24 h incubation), surfaces were rinsed with PBS, and cells fixed in a 10% formaldehyde solution, permeabilized, and blocked in 1% (w/v) BSA/PBS. hMSCs were stained with 1:150 mouse anti-vinculin and 1:500 phalloidine-rhodamine in 1% (w/v) BSA/PBS for 1 h at 37 °C. After rinsing samples 3 × 5 min in 0.5% (v/v) Tween-20/PBS, 1:50 biotinylated anti-mouse secondary antibody (Vector Laboratories) was added in 1% (w/v) BSA/PBS and incubated again at 37 °C for 1 h. After washing, 1:50 FITC‐conjugated streptavidin (Vector Laboratories) was added and incubated for 30 min at 4 °C. Finally, disks were washed and mounted using Vectashield mountant with DAPI nuclear stain (Vector Laboratories). All reagents are from Sigma-Aldrich, unless otherwise stated. Images from the immunostaining experiments were analyzed with the Fiji/Image-J package^53^. A self-made macro was used to quantify DAPI-stained nuclei, while characterization of cell-projected area, shape and focal adhesion length was done manually, using about 200 values per condition.

### PCR analysis

After culturing cells for 21 days on Ti disks, total RNA was extracted using the RNeasy micro kit (Qiagen, Hilden, Germany) according to manufacturer instructions. A NanoDrop spectrophotometer (Thermo Scientific, Waltham, MA, USA) was used to quantify total RNA. RNA was reverse transcribed to cDNA using the QuantiTect Reverse Transcription Kit (Qiagen). Real time qPCR was carried out to quantify the expression of osteocalcin (OCN) and osteopontin (OPN) genes with QuantiTect SYBR Green RT-PCR Kit (Qiagen) on a 7500 Real-Time PCR system (Applied Biosystems, Foster City, CA, USA). Primer sequences are reported in Table 2. Melt curve analysis was used to validate the primer sequences for the genes. Gene expression was normalized to GAPDH expression, which was chosen as the housekeeping gene, and analyzed with the 2^−*DDCT*^ method.

**Table 2:** PCR primer sequences.

Target Gene	Forward Sequence	Reverse Sequence
GAPDH	GTCAGTGGTGGACCTGACCT	ACCTGGTGCTCAGTGTAGCC
OPN	AGCTGGATGACCAGAGTGCT	TGAAATTCATGGCTGTGGAA
OCN	CAGCGAGGTAGTGAAGAGACC	TCTGGAGTTTATTTGGGAGCAG

### Bacterial adhesion

*P. aeruginosa* ATCC 27853 was grown aerobically for 16 h in 10 mL Luria-Bertani broth (LBB) in a 37 °C shaker incubator set at 220 rpm. The bacterial suspension was then diluted in LBB to OD_600_ 0.1 and further incubated until mid-exponential phase was reached. At this time bacterial cells were harvested by centrifugation (7 min, 5000 g), washed twice in 10 mM Tris-HCl buffer (2-amino-2-(hydroxymethyl)-1,3-propanediol, adjusted to pH 7 with hydrochloric acid), and suspended in Tris-HCl buffer to OD_600_ 0.3 (approx. 10^7^ cfu/mL).

The test surfaces and controls were placed into a 12-well microtiter plate and submerged in 2 mL of bacterial suspension. Plates were incubated for 1 h at 37 °C under static conditions. After incubation, surfaces were rinsed to remove non-adherent bacteria by passing back and forth five times in a Universal container containing Tris-HCl buffer, repeated three times in total.

Following rinsing, 1 mL of Live/Dead® BacLight™ bacterial viability stain (Invitrogen) was applied to the surfaces (as per manufacturers’ instructions) and incubated in the dark for 15 min at RT. Surfaces were then rinsed in Tris-HCl buffer as described above to remove excess stain. Surfaces were maintained in 1 mL of Tris-HCl buffer, and bacterial adhesion and viability was visualized by fluorescence microscopy. Image J software was used to calculate the number of cells with intact membranes (SYTO 9, green) and the number of cells with damaged membranes (propidium iodide, red) based on 5 images per surface. Three independent experiments were performed and from these the average % kill was determined by (no. of damaged cells/total no. of cells) * 100 ± SEM.

### Statistical analysis

Statistical comparisons were based on analysis of variance (ANOVA) with Tukey post hoc test for pairwise comparisons and non-parametric Mann-Whitney test. Results are presented as mean + SEM.

## Electronic supplementary material

Supplementary Information
