# Supplementary material for: Towards the cell-instructive bactericidal substrate: exploring the combination of nanotopographical features and integrin selective synthetic ligands
Source: Sci Rep. 2017 Nov 27;7:16363. doi: 10.1038/s41598-017-16385-3 (PMC5703844; doi:10.1038/s41598-017-16385-3)

SUPPLEMENTARY INFORMATION

Towards the cell-instructive bactericidal substrate: exploring the combination of nanotopographical features and integrin selective synthetic ligands

R. Fraioli, P.M. Tsimbouri, L.E. Fisher, A.H. Nobbs, B. Su, S. Neubauer, F. Rechenmacher, H. Kessler, M.P. Ginebra, M.J. Dalby, J.M. Manero, C. Mas-Moruno

Table of contents

Supplementary Table S1 Page S2

Supplementary Table S2 Page S2

Supplementary Figure S1 Page S3

Table S1. Additional topographical values.

	Roughness parameters [a]					
Surface	R_a *(nm)*	R_{ku}	R_p *(nm)*	R_q *(nm)*	R_{sk}	R_z *(nm)*
FLAT	36 ± 5	3.3 ± 0.5	187 ± 98	45 ± 5	-0.62 ± 0.38	291 ± 86
FINE	84 ± 7	9.0 ± 3.1	1475 ± 718	117 ± 8	0.16 ± 0.71	1735 ± 270
COARSE	512 ± 105	11.9 ± 4.4	7063 ± 2270	740 ± 169	2.33 ± 0.54	8186 ± 2232

[a] R_a, average roughness; R_{ku}, sharpness of the surface height distribution or surface kurtosis; R_p, largest peak height; R_q, root mean square; R_{sk}, asymmetry of the height distribution or surface skewness; R_z, difference between the highest and lowest peaks.

Table S2. Chemical Composition (atomic percentages) of the biofunctionalized surfaces and uncoated controls by XPS.

	C 1s	N 1s	O 1s	Si 2p	Ti 2p	Normalized N(a)	Ratio(b)
FLAT	44.1	1.2	40.9	0.2	13.6		
+ P	64.0	9.6	22.1	0.9	3.4		
+ V3	59.6	8.1	26.5	0.1	5.8	0.63	1.4
+ 51	62.1	7.8	25.0	0.0	5.2	0.46	
FINE	29.26	0.52	50.22	0.49	19.5		
+ P	57.26	10.86	25.18	2.35	4.34		
+ V3	51.74	8.95	31.05	0.04	8.21	0.69	1.2
+ 51	53.06	9.7	30.1	0.11	7.04	0.57	
COARSE	19.28	0.47	56.73	0.85	22.67		
+ P	50.06	10.91	29.31	2.36	7.36		
+ V3	55.44	11.71	26.92	0.15	5.77	0.90	1.6
+ 51	45.44	9.48	34.68	0.21	10.19	0.56	

a) Percentage of the N 1s signal normalized to the number of N in each peptidomimetic.

b) Ratios of V3/51 expressed as: *normalized N content (V3) / normalized N content (51)*

Figure S1. Cell projected area on the uncoated FLAT, FINE and COARSE surfaces.

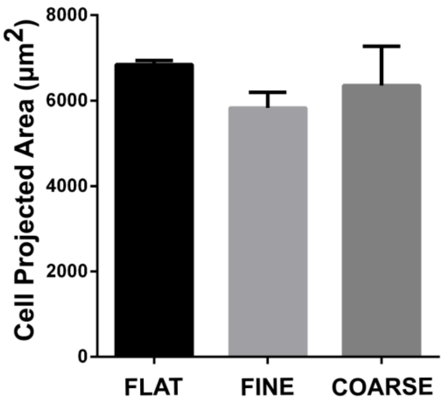

Supplement: Supplementary file 1 — Supplementary Information [file 41598_2017_16385_MOESM1_ESM.pdf]
